# A trait spectrum linking nitrogen acquisition and carbon use of ectomycorrhizal fungi

**DOI:** 10.1111/nph.70129

**Published:** 2025-04-05

**Authors:** Karolina Jörgensen, Karina E. Clemmensen, Petra Fransson, Stefano Manzoni, Håkan Wallander, Björn D. Lindahl

**Affiliations:** ^1^ Department of Soil and Environment Swedish University of Agricultural Sciences Box 7014 750 07 Uppsala Sweden; ^2^ Department of Forest Mycology and Plant Pathology Swedish University of Agricultural Sciences Box 7026 750 07 Uppsala Sweden; ^3^ Department of Physical Geography and Bolin Centre for Climate Research Stockholm University 106 91 Stockholm Sweden; ^4^ Department of Biology Lund University Sölvegatan 37 223 26 Lund Sweden

**Keywords:** carbon use efficiency, ectomycorrhizal exploration types, extraradical mycelium, functional traits, fungal ecology

## Abstract

Trait spectra have been used in various branches of ecology to explain and predict patterns of species distributions. Several categorical and continuous traits have been proposed as relevant for ectomycorrhizal fungi, but a spectrum that unifies co‐varying traits remains to be established and tested. Here, we propose a nitrogen acquisition and carbon use trait spectrum for ectomycorrhizal fungi in nitrogen‐limited forests, which encompasses several morphological, physiological, and metabolic traits. Using a simple stoichiometric model, the trait spectrum is linked to the concept of apparent carbon use efficiency and resolves the contradiction that species with high supply of host carbon can maintain nitrogen transfer despite building large mycelial biomass. We suggest that ectomycorrhizal fungal species are distributed along this spectrum, with lifestyles ranging from ‘absorbers’ with a niche in high productive forests with high availability of soluble nitrogen to ‘miners’ with the ability to exploit organic matter in forests with low nitrogen availability. Further, we propose ways to test the outlined trait spectrum empirically.

One objective in ecology is to find relations among organismal traits, species distributions, and various ecological roles that can be broadly generalised. These generalisations allow for navigating the complexity of observations and provide opportunities to predict changes in ecosystem functions. Such links may be particularly interesting when response traits, that is traits that determine environmental filtering of species, are correlated with effect traits, that is traits that determine how the presence of species influences the ecosystem (Violle *et al*., 2007). If environmental perturbations lead to significant and persistent changes in communities based on the response traits of species, associated changes in effect traits may lead to altered ecosystem functionality (Allison & Martiny, 2008).

An early trait spectrum, developed in animal ecology, was the K‐r strategist's framework (MacArthur & Wilson, 1967), which describes adaptations of reproduction strategies to different types of selection pressures, focusing on establishment, population densities, and competitive strength of species. Based on similar ideas about species distributions along continuous trait axes, Grime (1974, 1977) developed a framework that describes three main ecological strategies of plants: competitive, stress tolerant, or ruderal. Within plant science, another widely adopted trait spectrum is the leaf economics spectrum (Wright *et al*., 2004), which unifies physiological and morphological traits along a common axis, spanning from plants with long lived, sturdy leaves and slow growth to plants with fast growth and larger, thinner leaves. These concepts are now being expanded to characterise life history strategies of soil microorganisms (e.g. the Yield, resource Acquisition, Stress tolerance framework; Malik *et al*., 2020), but corresponding theories for soil fungi remain to be established and tested.

Calls for finding unifying traits among fungi have been made (Cooke & Rayner, 1984; Chagnon *et al*., 2013; Crowther *et al*., 2014; Koide *et al*., 2014; Treseder, 2023), and until the development of DNA‐ and RNA‐based molecular methods, trait‐based studies have largely focused on fruit bodies or morphological attributes of the mycelium. There have been previous suggestions for continuous trait spectra among ectomycorrhizal fungi, with an early attempt made by Mason *et al*. (1982), who described ectomycorrhizal fungi as early pioneers or late stage successors. This attribution referred to the ability of fungi to establish rapidly on roots of planted trees, or whether they became more frequent only as trees grew older. Leake & Read (1997) argued that mycorrhizal fungi vary in their capacity to mobilise nitrogen (N) from differently accessible sources. Later, the concept of ‘exploration types’ (Agerer, 2001), based on morphological traits of emanating mycelium from ectomycorrhizal root tips, was developed. The extent and mode of exploration into the soil matrix has been proposed to reflect other ecophysiological traits of ectomycorrhizal fungi, for instance their response (tolerance or preference) to N supply (Lilleskov *et al*., 2019). Ectomycorrhizal fungal species are often described as either nitrophobic, nitrotolerant, or nitrophilic with regard to shifts in relative abundances in response to variation in (anthropogenic) inorganic N‐supply (Lilleskov *et al*., 2011; van der Linde *et al*., 2018). Exploration types have also been linked to patterns of N allocation between extraradical mycelia and hosts (Hobbie & Agerer, 2010), and with the capacity to exploit organic substrates (Lilleskov *et al*., 2002; Argiroff *et al*., 2022).

The usefulness of mycorrhizal mycelial exploration types as predictors of soil colonisation and biomass was recently questioned, because genera expected to have extensively proliferating extraradical mycelium (medium and long distance types) were not consistently efficient soil colonisers (Jörgensen *et al*., 2023). Moreover, the response of ectomycorrhizal fungi to externally added N (fertilisation, atmospheric deposition) is commonly negative (Lilleskov *et al*., 2011; van der Linde *et al*., 2018; Jörgensen *et al*., 2022). However, in strongly nutrient‐limited systems, N additions can stimulate ectomycorrhizal fungi (Clemmensen *et al*., 2006; Högberg *et al*., 2021). Thus, N responses, and whether a species would be perceived as nitrophobic or nitrophilic/nitrotolerant, seem to be context dependent (Jörgensen *et al*., 2024). Since the realised niche of organisms depends on multiple traits, we believe that an ecophysiological trait spectrum could unify inconsistencies within and among currently used categorical traits related to N acquisition and carbon (C) use (i.e. exploration types, N response, and hydrophobicity). Here, we discuss different ectomycorrhizal fungal traits and how they may co‐vary to form a trait spectrum with lifestyles ranging from nutrient ‘absorbers’ to ‘miners’. Further, we propose that ‘apparent C use efficiency’ (Manzoni *et al*., 2018) could be an integrated metric synthesising important ecophysiological traits of ectomycorrhizal fungi, and so may provide an axis to characterise species along the absorber‐to‐miner spectrum. Currently, these ideas build on indirect, community‐level mycelial properties and hypothetical reasoning, and remain to be tested using empirical data on traits of ectomycorrhizal mycelia, which are inherently difficult to study in isolation. The trait spectrum we propose is particularly relevant to forests where both ectomycorrhizal fungi and hosts are limited by N, conditions common in boreal conifer‐dominated systems (Högberg *et al*., 2021), and it is focused on the interactions of C utilisation and N uptake and transport during the active growing phase of the extraradical mycelium after a major disturbance. After disturbances resulting in dieback of extraradical mycelium (e.g. drought or freezing), mycorrhizal fungi may mainly reside on the root tips or as cords, and more diffuse extraradical mycelium has to re‐establish, with mycelial proliferation strongly dependent on C‐use efficiency (CUE). Other traits, such as phosphorus uptake, drought tolerance, dispersal and root colonisation, micro‐habitat preference, or non‐nutritional benefits are beyond the scope of this Viewpoint, but would surely be important to describe the full trait‐space of ectomycorrhizal fungi.

## Growth properties

Ectomycorrhizal fungi rely on photosynthetic C from their host plants, which is used to produce mycorrhizal structures and extraradical mycelial biomass (Saikkonen *et al*., 1999; Lilleskov *et al*., 2011; Moeller *et al*., 2014; Fernandez *et al*., 2017; Defrenne *et al*., 2019; Pellitier & Zak, 2021; Suz *et al*., 2021). In this context, genera supposed to have large amounts of extraradical mycelia, for instance *Suillus* (long distance), *Piloderma*, and *Cortinarius* (medium distance), would be particularly C‐demanding (Lilleskov *et al*., 2019), while genera with less prolific mycelia (contact and short distance) would be less demanding in terms of C (Fernandez *et al*., 2017). In support of this hypothesis, long‐ranging, cord‐forming ectomycorrhizal fungi were more sensitive to reduced C allocation (induced by defoliation) than species with less extensive mycelia (Saikkonen *et al*., 1999). Similarly, drought‐induced reduction in photosynthesis rates favoured short‐distance, low biomass genera over genera with more extensive mycelia (Castaño *et al*., 2018). However, direct measurements of mycelial proliferation from root tips into soil do not support this picture since exploration types were not consistent predictors of biomass accumulation in ingrowth bags (Jörgensen *et al*., 2023).

The fungal demand of host C does not only depend on the growth of extraradical mycelial biomass alone but also depend on fungal CUE (Eqn 1), that is the proportion of assimilated C (uptake; $U_{C}$) that is incorporated into biomass (growth; *G*) (Manzoni *et al*., 2018; Hagenbo *et al*., 2019),

(Eqn 1) $$\mathrm{CUE}=\frac{G}{U_{C}}$$

We argue that CUE may be used as an emergent trait to disentangle different ecological strategies of ectomycorrhizal fungi. High CUE can be associated with high production of extraradical mycelium and low respiration and exudation in relation to C supply from the host, whereas low CUE can be associated with low extraradical growth and a large fraction of host C respired or exuded. There are indications that some ectomycorrhizal fungi may have very low CUE, suggesting large variation within the guild (Horning *et al*., 2023). Across ectomycorrhizal species, a high C demand could, thus, result from either low CUE or from high growth rate.

Mycelial biomass accumulation also depends on the rate of biomass turnover, that is the mortality of the mycelium (Clemmensen *et al*., 2013; Ekblad *et al*., 2013; Hagenbo *et al*., 2017, 2018). Species with rapid turnover (i.e. high mortality; *M*) of extraradical hyphae would require more C than species with low mycelial turnover to attain the same standing biomass and relative abundance in the community. At the community level, *in situ*, it may be difficult to differentiate between low CUE and rapid biomass turnover, as CUE is estimated from net biomass growth, which in turn is affected by mortality. The two concepts may, instead, be unified into the concept of ‘apparent CUE’ (CUE_A_; Eqn 2), which is the net increase in biomass (i.e. G−M) per acquired C ($U_{C}$), subjected to losses by respiration, exudation, and turnover by mortality, over a specified time scale (Manzoni *et al*., 2018).

(Eqn 2) $${\mathrm{CUE}}_{A}=\frac{G-M}{U_{C}}=\mathrm{CUE}-\frac{M}{U_{C}}$$

**Fig. 1:**
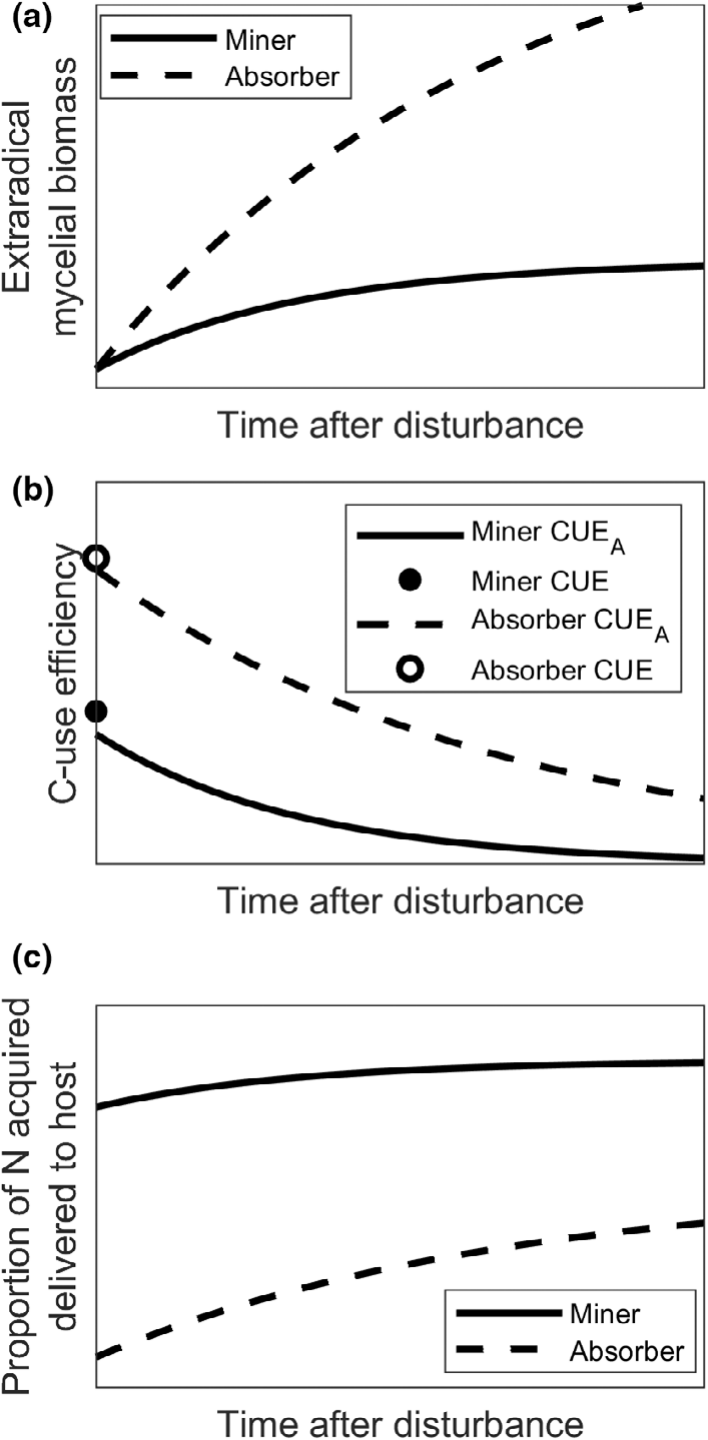
After a major disturbance associated with high mortality of extraradical mycelium, such as drought or freezing, the formation of new extraradical biomass depends on whether a species has absorber or miner traits. More rapid accumulation of extraradical biomass would be associated with higher apparent C‐use efficiency (CUE) of the absorbers (a). Apparent CUE would be higher during the initial phase of regrowth and decrease as biomass and associated mortality increase (b). The lower apparent CUE (higher mortality) of miners and higher capacity to acquire organic N relative to absorbers results in a higher share of acquired N delivered to the host (c). C, carbon; N, nitrogen.

Eqn 2 shows that CUE determines the potential growth rate of an ectomycorrhizal fungus, but the realised growth may be lower due to mortality, which in turn may be affected by the environment. A trait spectrum could, thus, range from species with high apparent CUE (minimal respiratory, mortality, and/or exudation losses) that require less host C per unit of mycelial biomass produced, compared to species with low apparent CUE (high respiratory losses and/or fast mycelial turnover) that require more host C to attain the same biomass in the community (Fig. 1a).

In the long term, under undisturbed conditions, apparent CUE becomes zero, because growth and mortality are approximately the same; that is $G\;\approx \;M\to {\mathrm{CUE}}_{A}\;\approx \;0$ and standing biomass stabilises at steady‐state. When biomass declines (e.g. due to disturbance, seasonal fluctuation, or antagonistic interactions), apparent CUE turns negative, because $G<M\to {\mathrm{CUE}}_{A}<0$. Therefore, it is meaningful to consider apparent CUE during the active growth period, when there is a net biomass accumulation and $G>M\to {\mathrm{CUE}}_{A}>0$. The dependence of apparent CUE on the time frame of observation requires some caution in its use as a fungal trait. It can provide useful information when ${\mathrm{CUE}}_{A}$ is estimated across fungal species but within the same time frame, when all species are in their active growing phase.

## Nitrogen acquisition properties

In exchange for host C, ectomycorrhizal fungi supply the host with N, which they either absorb from the soil solution or mine from solid organic matter by exuding extracellular enzymes and/or using oxidative mechanisms (Read, 1991; Lindahl & Tunlid, 2015; Tunlid *et al*., 2022). The dissolved N forms include inorganic ammonium (NH_4_^+^) and nitrate (NO_3_^−^), but amino acids also constitute a large part of the N supply in forest soils (Kranabetter *et al*., 2007; Inselsbacher & Näsholm, 2012). Some ectomycorrhizal fungal genera, such as *Amphinema*, *Laccaria*, and *Thelephora*, seem to specialise in efficient uptake of mineralised N forms, while others, such as *Cortinarius*, have a lower capacity to take up inorganic N (Kranabetter *et al*., 2015). Efficient uptake of soluble N with minimal leaching losses should depend on the capacity to build a dense extraradical mycelium (Hobbie & Agerer, 2010; Bahr *et al*., 2015), which in turn benefits from high CUE (for a given amount of host C over a given time span). The ability to mobilise tightly bound organic N from organic matter also differs among ectomycorrhizal fungi. While the majority of ectomycorrhizal fungi lost the capacity for organic matter decomposition when they evolved from saprotrophic ancestors (Kohler *et al*., 2015), some have retained a capacity to produce manganese peroxidases (Bödeker *et al*., 2014) or use other oxidative mechanisms (Tunlid *et al*., 2022). This activity is probably pivotal for N mobilisation from low‐quality organic matter in nutrient‐poor environments (Bödeker *et al*., 2014; Shah *et al*., 2016; Nicolás *et al*., 2019; Clemmensen *et al*., 2021; Lindahl *et al*., 2021; Pellitier & Zak, 2021; Argiroff *et al*., 2022). Another possible mechanism by which ectomycorrhizal fungi could mediate nutrient cycling is through priming of saprotrophic decomposers (Mayer *et al*., 2021). Mobilisation of N from recalcitrant organic matter, either by enzymatic and non‐enzymatic decomposition or by mycorrhizal priming of saprotrophs, is likely to be demanding in terms of host C, and a trade‐off between exploitation of recalcitrant resources and CUE seems likely (Shimizu *et al*., 2005; Chakrawal *et al*., 2024). Therefore, the mode of N acquisition could be aligned with CUE, with efficient species (high apparent CUE) being more successful in attaining high biomass and acquiring soluble N, while a low apparent CUE would provide energy for mobilisation of organic N, since production and maintenance of extracellular enzymes is metabolically demanding (Fig. 1b).

## Morphological and physiological properties

One adaptation for maximised uptake of dissolved nutrients is to grow extensive, diffuse extraradical mycelia, to ensure a high surface area in contact with the soil solution (Bahr *et al*., 2015; Almeida *et al*., 2022). By contrast, ectomycorrhizal fungi with high capacity for oxidative mobilisation of nutrients from organic matter often form mycelial ‘cords’ (Clemmensen *et al*., 2015; Argiroff *et al*., 2022). Cords consist of aggregated, vacuolised, or sometimes even dead, hyphae and have the capacity for high rates of apoplastic nutrient transport (Cairney, 1992). A differentiated mycelial morphology with cords is often associated with dynamic growth patterns governed by source–sink dynamics, in which fungi sacrifice senescing parts of their mycelium to internally recycle and redirect nutrients to actively growing mycelial fronts, colonising discrete resource patches (Lindahl & Olsson, 2004). Such dynamic mycelial behaviour has mainly been described among saprotrophic fungi, where it enables more efficient use of limiting nutrient resources (Boddy, 1999), but cord‐forming ectomycorrhizal fungi display the same behaviour (Finlay & Read, 1986; Leake *et al*., 2001; Donnelly *et al*., 2004). The ability to form cords may have been retained by some ectomycorrhizal taxa as they evolved from saprotrophic ancestors, increasing their fitness in strongly nutrient‐limited systems. For example, the extraradical mycelium of *Cortinarius* species resemble mycelia of related, saprotrophic *Hypholoma* and *Stropharia* species in the order Agaricales, and the excessive cord systems of *Suillus* species may be related to those of saprotrophic *Serpula*, both belonging to the order Boletales. Cord formation is a beneficial strategy for producing and maintaining long‐lived (perennial) mycelia, since the risk of disruption of mycelial integrity, for example by grazing, is smaller for aggregate mycelial structures than for fine hyphae (Boddy *et al*., 2009). The cords are usually hydrophobic, which improves their capacity for rapid, apoplastic nutrient transport (Cairney, 1992). Further, they can affect the way the mycelium interacts with soil organic matter (Unestam & Sun, 1995; Almeida *et al*., 2022). Thus, formation of hydrophobic cords could be a strategy to efficiently allocate mycelial growth to patchy solid organic resources and subsequently export mobilised nutrients to host roots (Cairney, 1992; Lindahl & Olsson, 2004).

## Nitrogen delivery to hosts

The exchange of C and N between hosts and ectomycorrhizal fungi is proposed to be regulated by source–sink relationships, and the trees are considered to be stronger C sources under conditions of low N availability (Bidartondo *et al*., 2001; Corrêa *et al*., 2011; Bunn *et al*., 2024). However, rapid growth of ectomycorrhizal mycelium in response to an ample supply of host C can lead to immobilisation of significant amounts of N in the mycelium (Colpaert *et al*., 1992; Corrêa *et al*., 2011), which could aggravate ecosystem N limitation (Näsholm *et al*., 2013). Cord formation, which entails vacuolisation, intrinsic turnover of senescent mycelium and redistribution of nutrients, results in a reduced mycelial N sink and could be a trait related to the ability to sustain N delivery to the host, despite N limitation of both partners in the symbiosis (Abuzinadah *et al*., 1986; Clemmensen *et al*., 2015). Högberg *et al*. (2021) observed a positive relationship between mycelial N content and N availability along a soil fertility gradient, in line with the idea that a lower N content of the mycelium (induced by vacuolisation) could decrease N retention in the mycelium and thereby increase excess N. Thus, ‘self‐decomposition’ in cord‐forming ectomycorrhizal fungi could result in a low apparent CUE and a slow net accumulation of mycelial biomass, making the mycelium a stronger N source for the host (Hagenbo *et al*., 2019). By contrast, high apparent CUE of ectomycorrhizal fungi would increase the N sink of the extraradical mycelium and lower the proportional N delivery to the host (Fig. 1c).

## Links between nitrogen fluxes and ectomycorrhizal traits

The ability of an ectomycorrhizal fungus to supply N to its host is determined by its traits in combination with soil N availability and C supply from the host. A simple stoichiometric model can illustrate these relations (model symbols are listed and explained in Table 1).

**Table 1:** Summary of symbols in model equations.

Abbreviation	Meaning	Unit
CUE	Carbon use efficiency	–
*U*_*C*_	Carbon transfer from host to mycorrhiza	g C m^−2^ d^−1^
*G*	Growth rate	g C m^−2^ d^−1^
CUE_A_	Apparent carbon use efficiency	–
*M*	Mortality rate	g C m^−2^ d^−1^
*U*_IN_	Inorganic nitrogen uptake rate	mg N m^−2^ d^−1^
*U*_ON_	Organic nitrogen uptake rate	mg N m^−2^ d^−1^
*r*_N_	N : C ratio of fungal necromass	mg N g C^−1^
*r*_F_	N : C ratio of fungal biomass	mg N g C^−1^
φ	Rate of production of excess N for transfer to host	mg N m^−2^ d^−1^

The model is based on the assumption of homeostatic fungal biomass with fixed N : C, $r_{F}=N_{F}/C_{F}$. From this assumption it follows that any net change in fungal N has to be equal to the net change in fungal C multiplied by $r_{F}$. Ectomycorrhizal fungal C increases by transfer of host C (subjected to losses by respiration and exudation) and decreases due to mortality. Fungal N can increase by uptake of inorganic or organic N, decrease due to mortality, and decrease when N is transferred to the host. Accounting for all these C and N fluxes, and recalling that they must be linked via the fungal N : C ratio, we can write,

(Eqn 3) $$\underset{\begin{array}{c} \text{inorganic}\;N\\ \text{uptake} \end{array}}{\underbrace{U_{\text{IN}}}}+\underset{\begin{array}{c} \text{organic}\;N\\ \text{uptake} \end{array}}{\underbrace{U_{\mathrm{ON}}}}-\underset{\begin{array}{c} \text{mortality}\\ N\;\text{loss} \end{array}}{\underbrace{Mr_{N}}}-\underset{\begin{array}{c} \text{excess}\;N\\ \text{for transfer}\\ \text{to host} \end{array}}{\underbrace{\phi }}=\underset{\begin{array}{c} \mathrm{net}\\ \text{change}\\ \text{in biomass}\;N \end{array}}{\underbrace{\left(G-M\right)r_{F}}}$$

where $U_{\text{IN}}$, $U_{\mathrm{ON}}$, ϕ, *G*, and *M* are the rates of inorganic N uptake, organic N uptake, production of excess N, fungal growth, and fungal mortality, respectively, and $r_{N}$ and $r_{F}$ are the N : C ratios of the fungal necromass and active fungal biomass, respectively. We also assume that senescing mycelium has a lower N : C ratio than the growing mycelium ($r_{N}<r_{F}$) to account for N retention during senescence. From Eqn 3, we can calculate the rate at which excess N that may be transferred to the host is produced,

(Eqn 4) $$\phi =U_{\text{IN}}+U_{\mathrm{ON}}-Mr_{N}-\left(G-M\right)r_{F}$$

For mycorrhizal fungi, the growth rate is defined as CUE times the rate of C acquired from the plants (denoted by $U_{C}$ to retain the same notation of Eqns 1, 2). Using this definition of growth rate and rearranging we find,

(Eqn 5) $$\phi =U_{\text{IN}}+U_{\mathrm{ON}}-\mathrm{CUE}\;U_{C}\;r_{F}+M\left(r_{F}-r_{N}\right)$$

Finally, it is convenient to normalise by the total N uptake rate $U_{\text{IN}}+U_{\mathrm{ON}}$ so that all rates are defined per unit N acquired from the soil and N excess is expressed as a proportion of the acquired N,

(Eqn 6) $$\begin{array}{rcl} \frac{\phi }{U_{\text{IN}}+U_{\mathrm{ON}}} & = & 1-\mathrm{CUE}r_{F}\frac{U_{C}}{U_{\text{IN}}+U_{\mathrm{ON}}}+\left(r_{F}-r_{N}\right)\frac{M}{U_{\text{IN}}+U_{\mathrm{ON}}}\\ & = & 1-r_{N}\frac{M}{U_{\text{IN}}+U_{\mathrm{ON}}}-{\mathrm{CUE}}_{A}r_{F}\frac{U_{C}}{U_{\text{IN}}+U_{\mathrm{ON}}} \end{array}$$

**Fig. 2:**
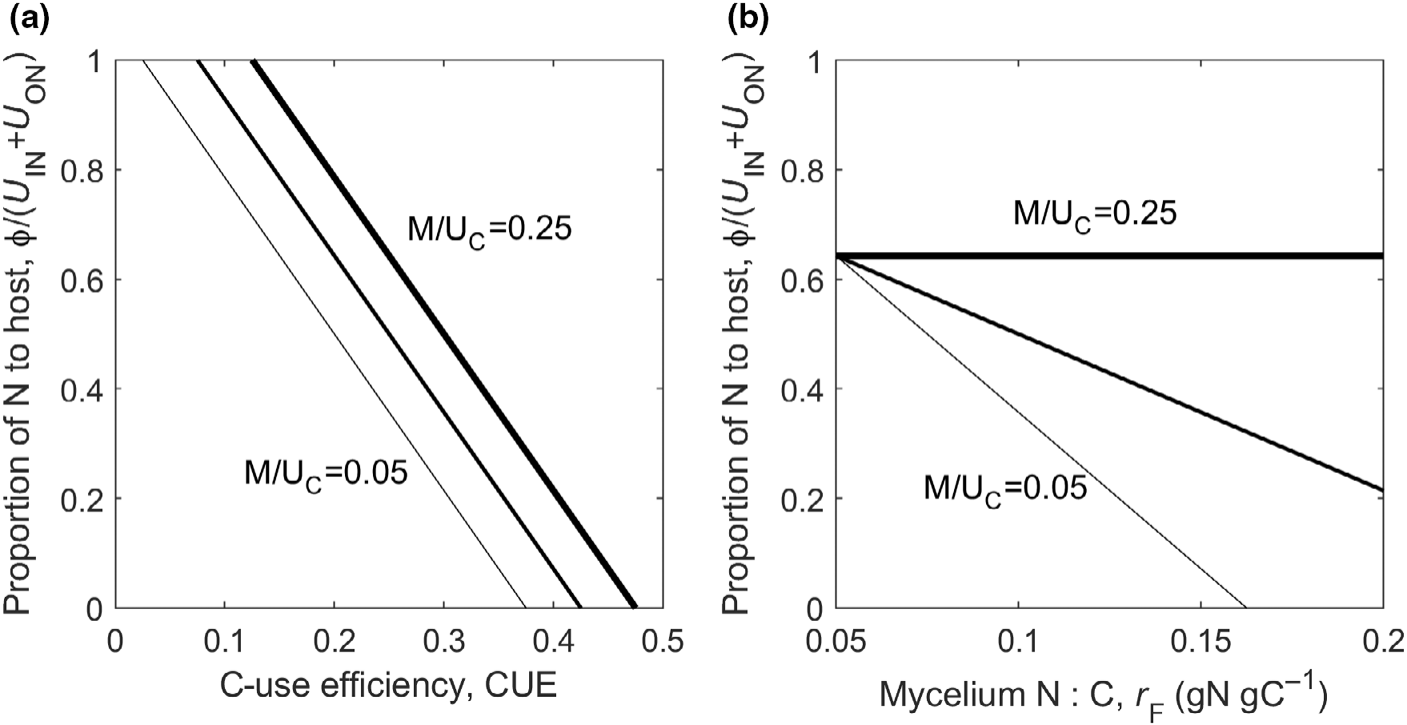
Proportion of N acquired from the soil by mycorrhiza that can be delivered to the host plant (Eqn 6), as a function of (a) C‐use efficiency (CUE) and (b) mycelium N : C ratio ($r_{F}$), at different levels of mycorrhiza mortality (*M*). Both N delivery rate and mortality rate are normalised by the rate of C transfer from the host to the mycorrhiza ($U_{C}$). This figure shows that N delivery to the host decreases with increasing CUE or mycelium N : C, and increases with higher mortality. Model parameters (when not varied as specified in the figure): CUE = 0.25, $r_{F}=0.1$ g N g C^−1^, $r_{N}=0.05$ g N g C^−1^, $U_{C}=1$ g C m^−2^ d^−1^, $U_{\mathrm{ON}}=0.025$ g N m^−2^ d^−1^, $U_{\text{IN}}=0.01$ g N m^−2^ d^−1^. C, carbon; N, nitrogen.

This equation shows how N delivery to the host plant is linked to the functional traits: CUE, N : C in active and senesced mycelium ($r_{F}$ and $r_{N}$), and mortality (*M*) (Fig. 2). In general, for a given inorganic N content (affecting to $U_{\text{IN}}$) and substrate N : C (affecting $U_{\mathrm{ON}}$), more N can be available for transfer to the host when one or more of the following occur: (1) CUE is low, (2) fungal N : C is low (i.e. fungal C : N is high), (3) *M* is high (only when fungi retain N at senescence; i.e. $r_{F}>r_{N}$), or (4) the N : C of senesced mycelium is low. The last equality in Eqn 6 links N delivery to the apparent CUE (Eqn 2). The interpretation of effects of ${\mathrm{CUE}}_{A}$ is similar to that of CUE – less efficient fungi can deliver more N to the host per N acquired.

## A functional trait spectrum from absorbers to miners

**Fig. 3:**
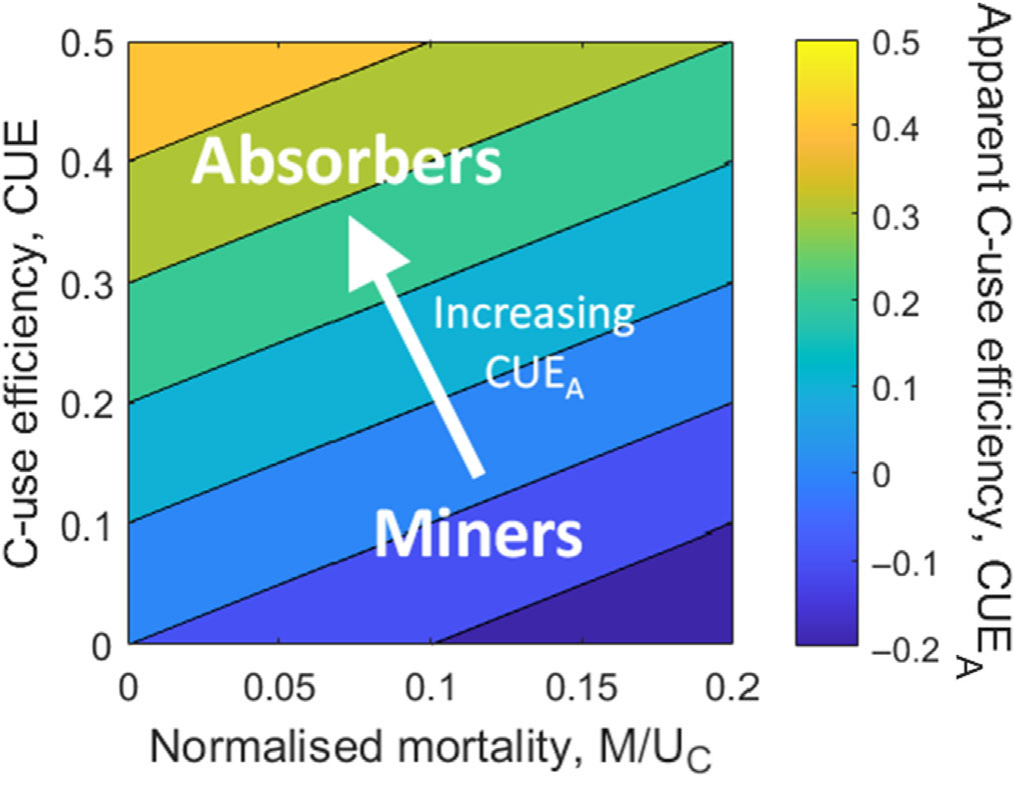
The C‐use efficiency (CUE) and mortality per assimilated C ($M/U_{C}$) of ectomycorrhizal fungi are related through the concept of apparent CUE (CUE_A_). Ectomycorrhizal fungi are proposed to be distributed along a gradient of CUE_A_ where ‘miners’ would have low apparent CUE (i.e. low CUE and/or high mortality) and ‘absorbers’ would have high apparent CUE (i.e. high CUE and/or low mortality). CUE_A_ shown as background shading is calculated from Eqn 2.

**Fig. 4:**
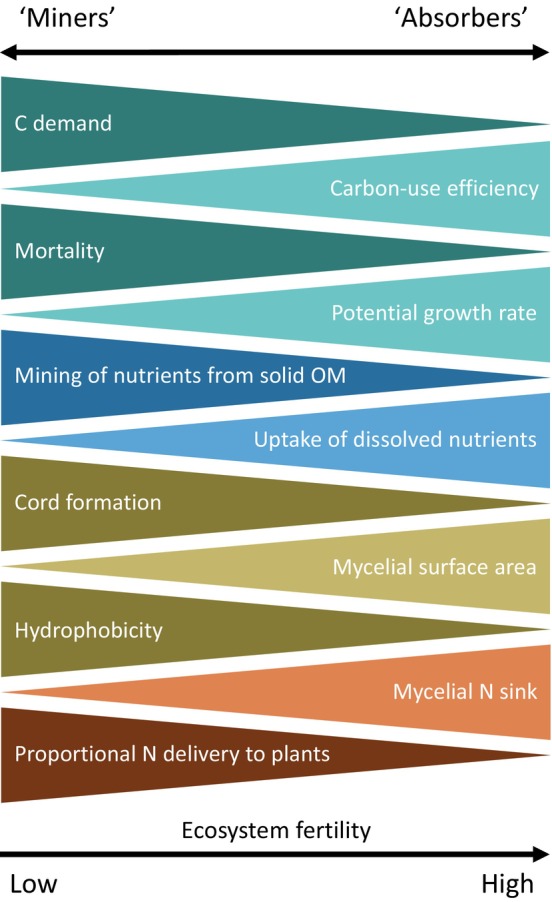
Co‐varying traits of ectomycorrhizal fungal ‘absorbers’ or ‘miners’. Triangle width corresponds to ‘strength’ of the trait. Colours denote category of traits related to growth, nutrient acquisition, morphology and physiology, and nitrogen (N) delivery to plants. OM, organic matter.

Considering all these traits together, we propose that ectomycorrhizal fungi in N‐limited forests may be organised along a functional spectrum from ‘absorbers’ to ‘miners’, where the ecological strategy is linked to apparent CUE during the growing period and regulated by the actual CUE and (self‐induced) mortality (Fig. 3). Further, the apparent CUE would be associated with other physiological and morphological traits (Fig. 4), which could explain species distributions across ecosystems with different degrees of N‐limitation. ‘Absorber’‐taxa would have high apparent CUE resulting in fast accumulation of biomass and their realised niche is primarily in forests with high supply rates of soluble N. By contrast, ‘miner’‐taxa would have a low apparent CUE, slow accumulation of biomass, and a realised niche in more strongly N‐limited forests, where they mainly acquire N from solid, recalcitrant organic sources. This framework could resolve contradictions and unify categorisations of ectomycorrhizal fungi based on exploration types, C demand, early vs late‐stage colonisers, hydrophobicity, and nitrophobicity.

‘Miners’ would use an ample supply of host C to generate energy and drive efficient and selective exploitation of patchy organic nutrients (Finlay & Read, 1986; Leake *et al*., 2001; Donnelly *et al*., 2004) rather than to rapidly produce dense and evenly distributed mycelial biomass. Moreover, by restricting the accumulation of N in mycelial biomass, either by slow growth or by rapid mycelial turnover, for example self‐decomposition associated with the formation of mycelial cords (Boddy, 1999; Clemmensen *et al*., 2015), ‘miners’ could deliver a larger fraction of acquired/released nutrients to their host (Abuzinadah *et al*., 1986). Dieback of exploratory hyphae could stimulate rapid turnover of necromass by free‐living decomposers, releasing nutrients for uptake (Mayer *et al*., 2021). By building hydrophobic mycelial cords that are less prone to grazing damage, they may, slowly but persistently, build large perennial mycelia with maintained connectivity, potentially resulting in large biomass in stable environments. Ample formation of long‐lived cords may, thus, explain the apparently slow mycelial turnover of ‘miners’ in old forests compared to young forests (Hagenbo *et al*., 2018), despite rapid turnover (i.e. mortality) and decomposition of exploratory hyphae (Dowson *et al*., 1989; Pritchard *et al*., 2008). Hence, it is likely that current methods to estimate turnover do not have the temporal resolution to capture the short‐term dynamics of the exploratory mycelia of ‘miners’. Examples of genera of the ‘miner’‐type would be *Cortinarius* and *Piloderma*, which often dominate ectomycorrhizal fungal communities and attain a high biomass in old, nutrient‐limited boreal forests (Twieg *et al*., 2007; Sterkenburg *et al*., 2015; Kyaschenko *et al*., 2017), where host C allocation to roots and mycorrhizal fungi is expected to be particularly high (Marshall *et al*., 2021). A slow net accumulation of biomass is supported by the low extraradical proliferation of these genera despite high abundance on roots in a one‐season incubation study (Jörgensen *et al*., 2023). In forests with larger amounts of easily available mineral N, where host C allocation belowground is low (Högberg *et al*., 2003; Marshall *et al*., 2021), the slow net growth of ‘miners’ will put them at a competitive disadvantage (Jörgensen *et al*., 2022), whereby they are perceived as nitrophobic. We, therefore, suggest that nitrophobicity is an indirect effect of the links between costly exploitation of organic nutrients, self‐induced mortality, low apparent CUE, and slow growth, rather than direct sensitivity to elevated inorganic N availability. Of course, such ‘indirect nitrophobicity’ may be combined with an enhanced tolerance for nitrogen‐poor and acidic habitats.

‘Absorbers’, which would have rapid proliferation of extraradical mycelium and, supposedly, high apparent CUE, may be better adapted to colonise new roots (Deacon *et al*., 1983) and immobilise soluble inorganic N, preventing leaching losses at minimal C supply from the host. Accordingly, *Amphinema*, *Thelephora*, and *Tylospora* had extensive extraradical proliferation in sand and soil patches during one growing season (Jörgensen *et al*., 2023). Moreover, across a chronosequence of *Pinus sylvestris* forests, Hagenbo *et al*. (2018) and Kyaschenko *et al*. (2017) found these genera to be associated with younger forests with higher inorganic N availability. In these forests, the CUE of the ectomycorrhizal fungal community was also higher than in older forests with lower N availability (Hagenbo *et al*., 2019). Accordingly, *Amphinema* increased in abundance towards the richer end of a southern boreal fertility gradient (Kranabetter *et al*., 2009), and *Paxillus* has been reported as tolerant to atmospheric N deposition (Lilleskov *et al*., 2011), as well as having a high growth to respiration ratio (Bidartondo *et al*., 2001). The high apparent CUE of ‘absorbers’ would imply that assimilated N, to a large extent, gets locked up in extraradical mycelium and that a minor fraction is delivered to the plant (Colpaert *et al*., 1992; Corrêa *et al*., 2011). While this may momentarily aggravate N limitation of trees (Näsholm *et al*., 2013), investment in ‘absorber’ genera could be a beneficial longer term strategy, as losses of N through leaching may be minimised (Bahr *et al*., 2015) and N‐immobilisation in mycelial biomass may suppress competing vegetation (Henriksson *et al*., 2021).

The position of an ectomycorrhizal fungus on the ‘miner’ to ‘absorber’ spectrum depends on the context. In the study by Jörgensen *et al*. (2023), *Piloderma* and *Cortinarius* did not attain high biomass in ingrowth bags despite high abundance on roots, indicating low apparent CUE. However, in another study (Jörgensen *et al*., 2022), *Piloderma* species responded negatively to N fertilisation in boreal forest in absolute terms, but still increased their relative share of the ectomycorrhizal fungal community, suggesting that they are ‘miners’, although less so than, for example, *Cortinarius* species. Moreover, *Tylospora* increased its relative abundance along an N availability gradient in Swedish forests subjected to elevated atmospheric N deposition (Jörgensen *et al*., 2024), yet *T. asterophora* was indicated as being nitrophobic across European (temperate) forests with very high atmospheric N loads (van der Linde *et al*., 2018). These observations support the usefulness of a continuous trait spectrum, which can potentially identify optima in species distributions across large geographical scales. It is also possible that species at either end of the spectrum present some traits but not others. For example, *Russula* species have many ‘miner’ traits but do not usually form cords, whereas *Amphinema* species have cords but otherwise mainly ‘absorber’ traits. Similarly, *Paxillus* species are cord‐forming and possess the capacity for Fenton chemistry (Nicolás *et al*., 2019) but they have a high capacity for inorganic N uptake (Nygren *et al*., 2008), and are generally considered to be nitrotolerant (Lilleskov *et al*., 2011). *Suillus* species also form long‐ranging cords, but are early colonisers of seedlings (Menkis *et al*., 2005) and are generally associated with younger forests (Hagenbo *et al*., 2018). Such inconsistencies are easier to handle in a multivariate and continuous trait index than in a strictly categorical framework.

In the study by Jörgensen *et al*. (2023), *Cenococcum* and *Hyaloscypha* species did not attain high biomass in ingrowth bags, suggesting that ectomycorrhizal ascomycetes may fall outside the ‘absorber’ to ‘miner’ spectrum, with little extraradical mycelium, and no mycelial cords. Possibly, these fungi have a high apparent CUE, yet little extraradical growth, implying that they have a particularly low demand for host C. Ectomycorrhizal ascomycetes may, thus, be of less value for the host in terms of nutrient acquisition, but could have other benefits, for example protection against pathogens or drought (Krywolap *et al*., 1964; Pigott, 1982; Jany *et al*., 2003; Fernandez & Koide, 2013; Gehring *et al*., 2017).

## Outlook

For traits to be ecologically informative, they need to be empirically underpinned. Currently, trait databases (e.g. Determination of Ectomycorrhizae (DEEMY) for exploration types, http://www.deemy.de/, Agerer & Rambold, 2004; FungalTraits, Põlme *et al*., 2020) are rather sparsely populated, and more data are needed to get an estimate of variability of traits at all taxonomical levels of the ectomycorrhizal fungal guild. We call for more empirical studies of soil exploration (extraradical proliferation and mycelial turnover) as well as physiological (hydrophobicity) and metabolic (CUE) characteristics (Table 2). For instance, a DNA‐based approach with ingrowth bags (Kjøller, 2006; Jörgensen *et al*., 2023) or abundance of ectomycorrhizal fungal taxa close to roots and in root‐free soil (Genney *et al*., 2006) could be useful to increase knowledge about the mode and rate of mycelial exploration. In addition, metatranscriptomics targeting genes involved in growth and respiration as a proxy for CUE (Barbi *et al*., 2020; Hasby *et al*., 2021) could be used to test the utility of the ‘absorbers’ to ‘miners’ trait spectrum.

**Table 2:** Proposed methods to measure traits suggested to describe the nitrogen (N) acquisition and carbon (C) use trait spectrum.

Variable	Approach	Examples of references
CUE; carbon delivery from host	Measure respiration and mycelial biomass in ingrowth bags; metatranscriptomics; isotopes	Hobbie *et al*. (2002); Hagenbo *et al*. (2019); Hasby *et al*. (2021)
N delivery to host	Isotopes; stable isotope natural abundance and experimental pulse‐trace labelling	Högberg *et al*. (2011, 2021); Pellitier *et al*. (2021)
Uptake of inorganic/organic N	Isotopes; experimental pulse‐trace labelling, enzyme assays, metatranscriptomics	Sterkenburg *et al*. (2019); Auer *et al*. (2024)
Mortality	Use ingrowth bags to study short‐term turnover of extraradical mycelia; metatranscriptomics	Wallander *et al*. (2013); Hagenbo *et al*. (2024)

CUE, C‐use efficiency.

The general applicability of the proposed trait spectrum has to be validated by testing: whether there is strong coordination of morphological, physiological, metabolic, and resource acquisition traits along the spectrum; and whether environmental niches of ectomycorrhizal fungal species are predictable based on their placements along the trait spectrum. If successfully validated, the spectrum can be used to characterise multiple aspects of ectomycorrhizal ecophysiology based on information on single or a few measurable fungal traits. This would be advantageous for soil C and nutrient cycling models, where mycorrhizal fungi are described by numerous parameters (corresponding to functional traits) that are now poorly constrained (e.g. Brzostek *et al*., 2014; Baskaran *et al*., 2017; Smith & Wan, 2019; Aas *et al*., 2024). We believe that the proposed trait spectrum can unify multiple major trait axes of relevance for understanding how ectomycorrhizal fungi interact with their tree hosts and soil processes in N‐limited forests.

## Competing interests

None declared.

## Author contributions

KJ wrote the first draft of the manuscript. SM drafted the models. KJ, KEC, PF, SM, HW and BDL took part in discussions and revised the text.

## Disclaimer

The New Phytologist Foundation remains neutral with regard to jurisdictional claims in maps and in any institutional affiliations.
